# Are Wearables Effective in LMICs?

**DOI:** 10.3389/phrs.2025.1607940

**Published:** 2025-05-09

**Authors:** Malika Sachdeva, Adeline Dugerdil, Antoine Flahault, Verena Carrara

**Affiliations:** Faculté de Médecine, Institut de Santé Globale, Université de Genève, Geneva, Switzerland

**Keywords:** wearables, smartwatches, non-communicable diseases, pedometers, LMICs

## Abstract

**Objectives:**

To provide an overview of research conducted in low- and middle-income countries that present the impact of wearables on non-communicable diseases’ health outcomes, and factors that influence the adoption of wearables in these countries.

**Methods:**

We conducted a scoping review following the Arksey and O’Malley framework. Two databases, PubMed and Web of Science, were searched for relevant articles published between January 2010 and June 2023. We included studies set in low- and middle-income countries that focused either on the impact of wearables on changes in body mass index, blood pressure, and glycated hemoglobin levels or on the factors influencing wearables adoption.

**Results:**

A total of seventeen studies were included in the review out of the 890 identified during the search. Our findings suggest that wearables might be effective in improving health outcomes, such as body-mass-index and diastolic blood pressure, especially when used in conjunction with other health interventions.

**Conclusion:**

Wearables such as pedometers can be effective in improving health outcomes. Their widespread use in low- and middle-income countries is limited by different factors, including technological literacy, network coverage, and cultural considerations.

## Introduction

Non-communicable diseases (NCDs) account for 75% of all deaths globally, leading to a high cost to healthcare systems [1]. Cardiovascular diseases (CVDs), cancers, chronic respiratory diseases, and diabetes are the most frequent NCDs. About 75% of all NCDs can be linked to modifiable risk factors, including the use of tobacco, unhealthy diet, insufficient physical activity, and harmful consumption of alcohol. Therefore, they are considered largely preventable [2]. Additionally, insulin resistance, high blood pressure, elevated triglycerides, obesity, and low levels of high-density lipoprotein cholesterol (HDL cholesterol), together called metabolic syndrome, are strong predictors in the development of CVDs and diabetes [2].

NCDs are increasingly seen as a burden in low- and middle-income countries (LMICs), where 82% of all premature deaths annually occur [1]. LMICs have the highest prevalence and mortality rates of CVDs [3]. By 2030, deaths from NCDs in LMICs are expected to increase to 41.8 million annually from 30.8 million in 2015, putting even more pressure on health systems that are grappling with limited resources [4].

According to the World Health Organization (WHO), digital technologies, such as the Internet of Things (IoT), remote monitoring, artificial intelligence, and smart wearables, amongst several others, hold significant promise in improving health outcomes, by enhancing diagnosis, treatment decisions, and self-management [5]. Wearables can be used to improve screening, prevention, and monitoring of NCDs since they allow for continuous recording of physiological data [6–8]. They range from increasingly popular consumer-grade smartwatches and activity trackers, such as Fitbits^®^, to medical-grade devices used to monitor heart rhythms, blood pressure, as well as gait, and nutrition. They are non-invasive or minimally invasive and can monitor health-related outcomes such as step counts, heart rate, blood pressure, and arrhythmias. WHO’s Global Strategy on Digital Health 2020–25 acknowledges the importance of addressing the major barriers faced by LMICs in implementing digital health technologies [5].

A recent umbrella review reported the clinical benefits of wearable trackers, especially in increasing physical activity - an important risk factor for NCDs. Consequently, the review described wearable trackers as a recommended tool [9]. However, a majority of studies included in the review were conducted in high-income countries, raising uncertainty about the generalizability of the findings to LMICs.

The aim of this scoping review, therefore, is to provide a comprehensive overview of research conducted in LMICs that presents the impact of wearables on health outcomes related to NCDs, as well as the factors that influence and impede the adoption of wearables in LMICs.

## Methods

The study followed the scoping review framework as proposed by Arksey & O’Malley [10] and adhered to the Preferred Reporting Items for Systematic Reviews and Meta-Analyses Extension for Scoping Reviews (PRISMA-ScR) guidelines.

### Eligibility Criteria

Qualitative and quantitative studies were included to answer the following two principal research questions.

1. How do wearables impact the health outcomes of NCDs in LMICs?
2. What are the principal factors influencing or impeding the adoption of wearables in the context of NCDs in LMICs?

Wearables were defined as any device that can be worn by the user and included watches, wristbands, textiles, or accessories, such as rings and glasses. LMICs were determined using the 2022 World Bank Classification [11].

CVDs and their associated major metabolic risk factors, namely obesity, diabetes, and hypertension, were the primary focus due to their widespread prevalence and significant impact on mortality rates, particularly in LMICs [1, 2].

Health outcomes were defined as any reported changes in quantifiable parameters of the selected NCDs, such as body mass index (BMI), blood pressure (BP), waist circumference, or blood glucose and cholesterol levels. Other key indicators for evaluating effectiveness (such as calories burned or number of steps) were reported if mentioned.

Factors influencing or impeding the adoption of wearables were any quantitative or qualitative factors reported by consumers, healthcare professionals, or other relevant stakeholders, including non-governmental organizations (NGOs) or health ministries’ staff.

The detailed inclusion and exclusion criteria are summarized in Table 1.

**TABLE 1:** Inclusion and Exclusion Criteria (Scoping review, low-and-middle income countries, 2010–2023).

Inclusion criteria	Exclusion criteria
Articles written in English or French	Articles written in languages other than English and French
Articles reporting results from low- and middle-income countries (based on the World Bank Classification, 2022)	Articles reporting results exclusively from high-income countries (based on the World Bank Classification, 2022)
Articles that either include: • Health outcomes of cardiovascular disease, diabetes, obesity, or hypertension Or • Articles that focus on the factors impacting the adoption of wearables	Articles that do not include • Health outcomes of wearables on cardiovascular disease, diabetes, obesity, or hypertension, or that do not focus on the factors impacting adoption of wearables • Studies that assessed nutrition, gait, or stroke rehabilitation were not included • Validity or reliability studies that focused only on the wearable’s accuracy were equally not considered for inclusion
Articles that focus on minimally invasive, wearable devices	Articles that focus on invasive devices or that do not focus on wearable devices. Articles that only focus on mobile apps or text-messaging
Randomized Control Trials, observational studies, qualitative studies, and systematic reviews	Study protocols, editorials, short reports, conference abstracts
Study population including adults, pregnant patients and children with the disease, or at risk of it For studies exploring the factors impacting adoption wearables, the study population included patients at risk or with the disease, as well as relevant stakeholders, such as healthcare professionals	Study population consisting exclusively of healthy adults or children
Articles published between the 1st January 2010 and 26 June 2023	Articles published before the year 2010 or after 26 June 2023

### Search Strategy

The search strategy was developed and conducted by the main author (MS). The search terms comprised of three key concepts:

a) Wearables, smartwatches, and other similar terms
b) Developing countries and LMICs (based on the World Bank Classification)
c) CVDs, diabetes, obesity, and hypertension.

To ensure the inclusion of all relevant literature since the launch of the first mainstream wearable device, the Fitbit Classic^®^, studies from January 2010 until June 2023 were searched in two databases, PubMed, and Web of Science because of their extensive use and global coverage. The complete search strategy and research queries can be found in Supplementary Material S1.

A secondary search using a backward and forward snowball strategy and hand-searching helped identify additional relevant studies.

If it was not possible to ascertain the study eligibility after the initial abstract and title screening, the manuscript was retrieved for a full-text reading.

### Study Selection

**FIGURE 1:**
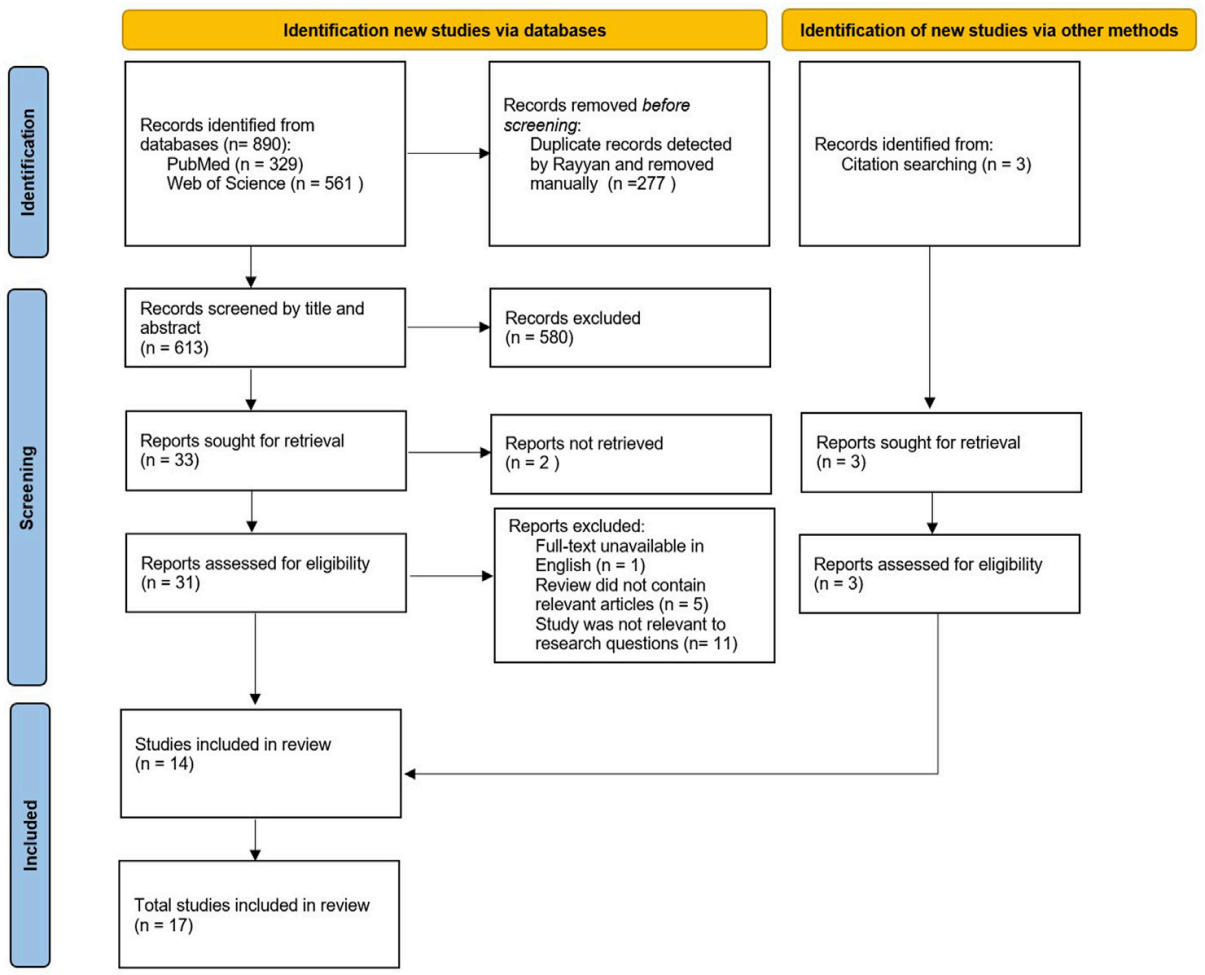
PRISMA chart depicting the study selection process (Scoping review, low-and-middle income countries, 2010–2023).

The final search was conducted on 26th June 2023 and yielded a total of 890 records from the two databases (Figure 1). The publications were then exported to Rayyan, a web-based tool for systematic reviews, which allows for deduplication, documentation of inclusion and exclusion reasons, as well as blinding selection decisions between reviewers [12]. Out of the 890 publications identified, 277 duplicates were removed, and 580 articles were excluded after an initial title and abstract screening since they did not fully meet the inclusion criteria. The remaining 33 articles were then assessed for eligibility after a full-text reading by two reviewers (MS and VC).

### Data Extraction and Analysis

Relevant information from all 17 studies was extracted from available full texts and reported into Excel 2023.

Four health outcomes commonly reported (BMI, glycated hemoglobin (HbA1c), systolic and diastolic blood pressure) were evaluated. Change-from-baseline and standard deviations were calculated from absolute values at baseline and at endpoint using the recommendations from Cochrane’s Handbook [13] unless already reported in the published results (Supplementary Table S2). To ensure a certain homogeneity, a 3-month endpoint was used for the present analysis as it was available for all, but one study [14]. Despite its shorter follow-up (2 months) this study by Shenoy et al. was nevertheless included in the analysis.

Standardized mean differences (SMD) and 95% confidence intervals were then calculated using RevMan (version 5.4.1) and forest plots were generated for each parameter. A SMD value of zero meant the changes in health outcomes were similar in both intervention and control groups. A negative SMD value suggested a stronger effect of the intervention on the changes in health outcomes compared to those observed in the control group. On the other hand, a positive SMD value indicated the changes observed in the intervention group were less than those observed in the control group. To interpret the strength of the effect, Cohen’s rules of thumb was used, where a SMD of ±0.2 represents a small effect, a SMD of ±0.5 represents a medium effect, and a SMD of ±0.8 represents a large effect [15, 16].

Risk of bias in individual RCTs was assessed using the Cochrane tools RoB 2 for randomized studies [17]. The likely extent of bias for each study was evaluated with a set of questions across different domains and reported following the GRADE guidelines (Supplementary Table S3) [18].

Factors evoked in the studies investigating barriers and facilitators impacting the adoption of wearables were summarized in a chart and described individually.

## Results

### Study Characteristics

Out of the 17 studies included, 10 were randomized controlled trials (RCTs). All studies were conducted in Asia, six (35%) in China, two (12%) studies each in Thailand, Turkey, and Cambodia, and the five (29%) remaining studies were set up in India, Pakistan, Indonesia, Sri Lanka, and Malaysia. All were conducted in middle-income countries: 65% (11/17) in upper-middle-income countries (UMIC) and the rest in lower-middle-income countries (LMIC). Our search did not find eligible studies set up in Africa or South America.

Nine RCTs looked at health outcomes of wearables; five (56%) reported changes in BMI, four (44%) changes in blood pressure (BP), and four (44%) measured changes in HbA1c. Six studies followed patients for 3 months, the remaining three had a follow-up of two, six, and 12 months, respectively.

Although pedometers were the most frequent wearables evaluated (6/9, 67%), study designs and the type of wearables exhibited heterogeneity (Table 2). Four of these studies reported the number of steps done using a pedometer, of which 2 reported it only in the intervention group and none reported number of calories burned (Supplementary Table S2).

**TABLE 2:** Study characteristics of the nine randomized-controlled trials investigating the impact of wearables on health outcomes (Scoping review, low-and-middle income countries, 2010–2023)^a^.

Author, year	Study country *(Income level)*	Type of wearable	Description of intervention and control	Duration (months)	Sample size	Study population
*Wearable and other intervention(s)* vs*. other intervention(s)*						
Gu, 2020 [19]	China *(UMIC)*	Pedometer	I: Pedometer + health education C: Health education-only	12 (*total*) *Follow up at 3 months*	I: 45 C: 45	Adults aged >60, who all had both hypertension and diabetes
Cayir, 2015 [20]	Turkey (*UMIC*)	Pedometer	I: Pedometer and low-calorie diet C: Low-calorie diet only	3	I: 45 C:39	Women >18 years of age with BMI >30kg/m2 without CVD, diabetes or medication
Li, 2021 [21],	China (*UMIC*)	Heart Rate Band	I: Received chest band, mobile application and telephone calls monthly to get supervised exercised C: Encouraged to exercise	3	I: 44 C: 41	Participants aged between 18 and 64 diagnosed with type 2 diabetes in the last 10 years, with access to smartphone
Yuting, [22]	China *(UMIC)*	Home-based BP monitor wearable wristband	I: wearable BP monitor, text-messaging, BP warning and home intervention C: Given information about hypertension and continued to receive usual care	3	I: 66 C: 68	Adults >40 years with a definite diagnosis of hypertension or being treated with hypertensive medication
Timurtas, 2022 [23]	Turkey *(UMIC)*	Wearable smartwatch	I: Received a smartwatch and created account on the DIABETEX platform, which could view their personal exercise program C: 3 sessions/week of supervised exercise ^b^	3	I: 28 C: 28	Adults between 30–65 years of age with, diagnosed with type 2 diabetes, possessing a smartphone
*Wearable-only* vs*. usual care*						
Omar, 2023 [24]	Malaysia *(UMIC)*	Pedometer	I: 12-week pedometer-based exercise C: Maintained their habitual lifestyle	3	I: 36 C: 34	Men aged 20–40 years with at least two or more CVD risk factors
*Wearable and other intervention* vs*. wearable-only*						
Chongthawonsatid, 2017 [25]	Thailand *(UMIC)*	Pedometer	I: Fitness program with supervised exercise once a week, health education and pedometer use C: Non-supervised use of pedometer	3	I: 30 C: 26	Adults between 30–65 years with pre-to-mild hypertension, not receiving any treatment for hypertension
Arovah, 2018 [26]	Indonesia *(LMIC)*	Yamax SW200 Pedometer	I: Received pedometer with text message support C: Received the pedometer only	6 (*total*) *Follow-up at 3 months*	I: 21 C: 22	Adults between 53–76 years with a clinically confirmed diagnosis of type 2 diabetes, owning a mobile phone, with familiarity with text messaging
*Wearable (+ other intervention)* vs*. usual care*						
Shenoy, 2010 [14]	India (*LMIC*)	Pedometer and Polar S410™ heart rate monitor	I: received pedometer and HRM, to achieve a target of 150-min/wk moderate intensity of aerobic physical activity C: continued medication as before	2	I: 20 C: 20	Participants diagnosed with type 2 diabetes, aged between 40 and 70 years, not taking insulin

^a^ I, intervention group; C, control group; UMIC, Upper-middle-income country; BMI, Body-mass-index; CVD, cardiovascular disease; LMIC, Lower-middle-income country.

^b^ The study consisted of three arms (wearable smartwatch, mobile application and supervised exercise). Results only pertaining to the wearable group and control were used since they were relevant to this study.

### Impact of Interventions on Various Health Outcomes

**FIGURE 2:**
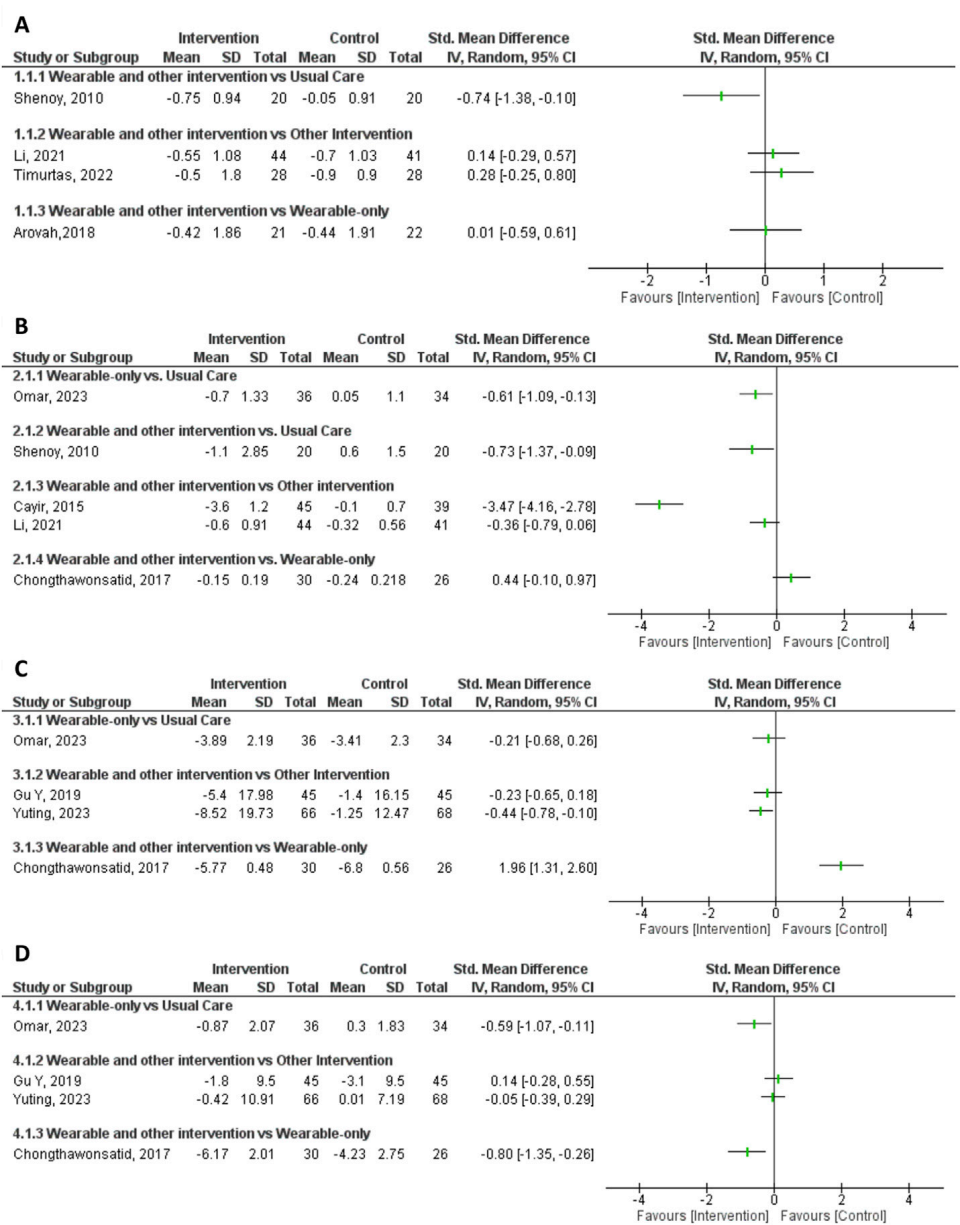
Forest plots illustrating the impact of the interventions on the four key health outcomes - **(A)**: HbA1c; **(B)**: Body-Mass Index; **(C)**: Systolic Blood Pressure; **(D)**: Diastolic Blood Pressure (Scoping review, low-and-middle income countries, 2010–2023).

The forest plots (Figures 2A–D) summarize the impact of the interventions on the four key health outcomes: changes in HbA1c levels, diastolic and systolic blood pressure values and body-mass index were the most commonly measured health outcomes and are detailed below.

#### HbA1c

Four of the five studies that enrolled participants with type 2 diabetes evaluated the changes in HbA1c. All four studies reported decreases in mean HbA1c levels post-intervention in both intervention and control groups, independently of their study designs (Figure 2A; Supplementary Table S2).

Only the study by Shenoy et al. reported a decrease in HbA1c significantly more important in the intervention group compared to the control (SMD = −0.74, 95% CI −1.4 to −0.09), demonstrating a large effect of their 150 min of aerobic activity a week with pedometer and heart rate monitor (HRM) intervention on HbA1c levels compared to regular medication only.

The three other studies found that HbA1c levels decreased slightly more in the control groups, indicating that the additional interventions weren't particularly effective. Specifically:

• Adding text messaging support to pedometer use showed no benefit (SMD = 0.01, 95% CI −0.59 to 0.61, Arovah et al.).
• Using a chest band, text messages, and a monthly call for supervised exercise didn’t help when both groups were already encouraged to exercise (SMD = 0.14, 95% CI −0.29 to 0.57, Li et al.).
• A smartwatch and personalized exercise program via a mobile app weren’t as effective as three supervised weekly exercise sessions (SMD = 0.28, 95% CI −0.25 to 0.80, Timurtas et al.).

Arovah et al. also reported results after 24-week of follow up and demonstrated long-term improvements. (SMD = −0.09, 95% CI –0.69 to 0.51, Arovah et al.).

Li et al. (SMD = 0.14, 95% CI −0.29–0.57), Timurtas et al. (SMD = 0.28, 95% CI −0.25–0.80), and Arovah et al. (SMD = 0.01, 95% CI −0.59–0.61), reported greater decreases in HbA1c for the control group, but these effects were relatively small, and their 95% confidence interval all included 0.

#### Body-Mass Index

Five studies examined the impact of wearables on BMI, and all of them reported decreases in mean BMI post-intervention (Figure 2B), independently of the target population and their clinical conditions.

Four studies included the evaluation of a pedometer to reduce weight in their study design. Cayir et al. evaluated the effect of proposing a pedometer in adult obese women on a low-calorie diet. This 3-month study showed the largest effect on BMI (SMD = −3.47). The effect was moderate-to-large (SMD = −0.73) in adults with type 2 diabetes when combining the use of a pedometer with a heart rate monitor and aiming at 150 min of weekly physical activity (Shenoy et al.). In Omar et al.’ study, adult men with two or more CVD risk factors were randomly assigned to use a pedometer or nothing. The wearable’s impact on participants' BMI was present, although moderate (SMD = −0.61). The beneficial effect of using a pedometer was also observed in the study of Chongthawonsatid et al. in which both groups of adults with mild hypertension received a pedometer.

The fifth study (Li et al.) included a multi-component intervention with a heart-rate band and a fitness app featuring an exercise plan proposed to adults with long-term type 2 diabetes. Although this intervention was more effective than that proposed to the control group, encouraged to exercise, the effect size was moderate (SMD = −0.36).

#### Systolic and Diastolic Blood Pressure (SBP and DBP)

Five studies included the evaluation of changes in mean blood pressure in their analysis (Figures 2C, D).

In adults with treated high blood pressure (HBP, Yuting et al.), the use of a wearable BP monitor, text messaging, and home intervention whenever necessary, had the biggest impact on SBP (SMD = −0.44, 95% CI −0.78 to −0.10) (Figure 2C). The impact of DBP was on the other hand minimal (Figure 2D). In study participants with mild untreated HBP (Chongthawonsatid et al.), the use of a pedometer, unsupervised or along with health education including weekly exercise had a large reduction in SBP and in DBP, although the size effect for SBP was in favor of unsupervised pedometer (SMD = 1.96, 95% CI 1.31–2.60) and for DBP in favor of that including weekly exercise (SMD = −0.80, 95% CI −1.35 to −0.26).

Two other studies evaluating the use of a pedometer (Shenoy et al. and Omar et al.) reported a moderate to large effect of the intervention on participants' DBP after 2 and 3 months of follow-up: SMD = −2.33, 95% CI −3.15 to −1.51 and SMD = −0.59, 95% CI −1.07 to −0.11, respectively. In older adults with dual HBP and diabetes assigned to either health education alone or coupled with the use of a pedometer (Gu et al.), the effect of the intervention was only observed after 12 months of follow-up (SMD = 0.14, 95% CI -0.28 to 0.55 at 3-month follow-up versus −0.18, 95% CI −0.60 to 0.23 at 12-month).

### Risk of Bias

The risk of bias in individual RCT was considered low or with moderate concerns across all 5 domains except in one study (Supplementary Table S3). Although studies were not blinded, analysis was mostly performed by staff independent of the clinical team. Loss to follow-up was minimal in most studies. In one study, the risk of bias was high concerning the randomization process and raised some concerns about bias in outcome measurement.

### Factors Influencing Adoption of Wearables in LMICs

A total of eight studies, including one RCT explored factors that influenced the adoption of wearables in LMICs. The studies are summarized in Table 3.

**TABLE 3:** Summary of studies exploring factors influencing the adoption of wearables in low-and-middle-income countries (Scoping review, low-and-middle income countries, 2010–2023).

Author, year	Study country *(Income level)*	Type of wearable	Study population
Liverani et al, 2022 [27]	Cambodia *(LMIC)*	Watch-type wearable	National and international stakeholders
Liverani et al., 2021 [28]	Cambodia *(LMIC)*	Watch-type wearable	Adults with and without diagnosed hypertension in urban and rural regions
Nadeem et al., 2021 [29]	Pakistan *(LMIC)*	Continuous Glucose Monitoring	All adults with a known diagnosis of type 1 or 2 diabetes
Arambepola et al., 2021 [30]	Sri Lanka *(LMIC)*	FitNLife activity monitor	1. Apparently healthy adults 2. Adults at risk of chronic NCDs 3. Community-based primary healthcare professionals
Zhang et al., 2020 [31]	China *(UMIC)*	Wearable BP Monitor	Adults >60 years with confirmed hypertension in the Xuanen area (rural region)
Yao et al., 2023 [32]	China *(UMIC)*	Wearable ECG device	General Practioners (GPs) in Sichuan Province
Huang et al., 2022 [33]	China *(UMIC)*	Wearable Sensors	Community dwelling older (>60 years of age) adults in Xuzhou
Lukkahatai et al., 2021 [34]	Thailand *(UMIC)*	Activity tracker (Garmin Vivofit)	Adults with diagnosed type 2 diabetes

**FIGURE 3:**
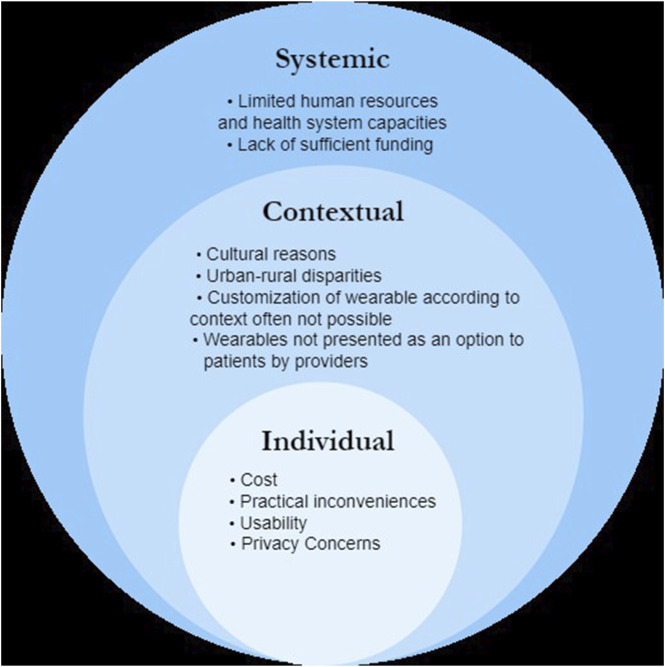
Summary of barriers to adoption of wearables in low-and-middle-income countries (Scoping review, low-and-middle income countries, 2010–2023).

The barriers to adoption of wearables highlighted in the studies above have been summarized in Figure 3.

Liverani et al. conducted two studies in Cambodia involving national and international stakeholders, as well as the general population. They revealed significant disparities between urban and rural populations concerning the adoption of wearables. Practical inconveniences associated with the use of wearables posed a significant barrier, particularly for farmers. Their studies highlighted that wearables were more suitable for young, urban residents, as they commonly use email accounts or mobile applications. Additionally, among poorer populations, the cost of wearables was cited as a major obstacle. Furthermore, the lack of shared Application Programming Interfaces (APIs) by private companies made customization of wearables challenging for specific contexts and languages.

Limited human resources and health system capacities were identified as crucial barriers to the widespread adoption of wearables for patient monitoring. Extensive training for health workers and potential challenges in integrating the increased volume of data generated by wearables into health information systems were also noted. Moreover, inadequate funding and a lack of coordination between donors made scaling up beyond pilot projects difficult.

The authors also observed that, culturally in Cambodia, the elderly are often cared for by their children, which led to wearables being perceived as less relevant in the community. Similarly, Huang et al. found in their study in Chinese community dwellings that participants with more children were less willing to choose smart senior care, including the use of wearable sensors in self-monitoring.

In their study based in Pakistan, Nadeem et al. identified the most common barriers to wearable technology adoption in diabetes management. The survey respondents reported that their diabetes care team had never discussed the use of wearables as a management option with them. Other common barriers included the patients' comfort with wearing the device as well as concerns about drawing attention from others.

Arambepola et al. reported in their study in Sri Lanka that feedback from activity monitors was the most frequently cited motivation for changing activity behaviors. However, participants required some guidance in interpreting the feedback initially. Similarly, Lukkahatai et al. found in their study in Thailand that participants who could see their steps significantly improved their step count and sleep duration compared to those who could not see their results.

Finally, in Yao et al.’s study on factors predicting general practitioners (GPs)’ adoption of wearable ECG devices in China, privacy concerns, social influences from peers and senior physicians, and price perception were identified as key factors influencing the adoption of wearable ECG devices.

## Discussion

This scoping review aimed to examine the impact of wearables on a set of health outcomes related to NCDs and to explore factors affecting their adoption in LMICs.

Seventeen studies met our research criteria, and all were implemented in middle-income countries, most (11 out of 17) being from upper-middle-income countries, and a notable concentration (6 out of 11) from China. None were from the African continent or South and Central America. This highlights a clear gap in research conducted in low-income and lower-middle-income countries, which has also been noted in previous studies [35, 36].

Our findings suggest that wearables might be effective in improving health outcomes, such as BMI and DBP, especially when used in conjunction with other health interventions. This highlights their potential to contribute to the reduction of the already high burden of CVDs in LMICs. However, their widespread use in LMICs is often limited by different factors, including technological literacy, network coverage, cost and cultural considerations.

### Impact of Wearables on Four Key Health Parameters

Our results align with previous studies indicating that wearable devices tend to be effective in increasing physical activity and supporting weight loss. However, they often have limited benefits in managing chronic diseases, and for other health outcomes, their effects tend to be small, often insignificant, and inconsistent [9, 37].

Out of the four studies that investigated the impact of wearables on HbA1c levels, only one study reported a significant improvement compared to the control group. Regarding systolic and diastolic blood pressures, significant improvements were found in three out of four studies for SBP and two out of four studies for DBP. These results are surprising, since controlling SBP is generally considered more difficult than DBP [38]. Finally, four out of five studies reported significant improvements in BMI.

### Factors Influencing the Adoption of Wearables in LMICs

The factors evoked in the studies included in this scoping review are quite similar to those that have already been reported. In a systematic review analyzing the barriers to the sustainability of digital health interventions in LMICs, factors such as limited technological literacy, cost, and limited human resources were evoked [39], which were similar to those that were reported in the studies included in this review.

Self-monitoring is not well-established in many LMICs, particularly among the elderly who are often cared for by family members [28, 33]. To address this cultural barrier children or younger caretakers could be involved by helping the elderly learn to use wearables, allowing them to self-monitor. Younger caregivers could also use these tools to monitor the elderly from a distance, therefore reducing the burden on family caregivers.

Additionally, one study revealed that patients often lacked sufficient information and support from healthcare professionals concerning wearable usage, emphasizing the need to first educate healthcare providers on the benefits of these wearables, so they can effectively communicate this knowledge to patients [29].

The feedback on activity levels provided by many wearables was cited as a common motivator to increase activity levels. However, customizing these products to local contexts and languages would enhance their usability and overall effectiveness. Furthermore, it was emphasized that wearables should be designed to withstand local working conditions. Therefore, by incorporating these features, wearables could be better suited to meet the needs of users in diverse LMIC settings [30].

### Strengths and Limitations

This scoping review has some limitations. Firstly, the search was conducted in two databases, PubMed and Web of Science; it was restricted to articles in French or English, with the search concluding in June 2023. Despite hosting a large body of literature worldwide, this restricted search may have left out relevant articles originating from countries underrepresented in the present manuscript, as well as studies with larger sample sizes. This field of research is rapidly expanding and future updates would certainly benefit from including regional databases and sources such as AJOL and LILACS. It could provide a more complete and a diverse perspective on factors influencing the adoption of wearables, and potentially could be challenging or refining our current conclusions. It would equally be important to consider the use of AI translation tools to include a larger body of manuscripts.

Secondly, despite a low to moderate risk of bias, the studies often had small sample sizes that could have limited the robustness of their results; and study designs were diverse, making direct comparisons and generalization challenging. In this context, subgroup analyses and meta regressions were not possible.

Another notable limitation was the relatively short study duration in most of the studies exploring the impact of wearables. Most of these studies had a study duration of only 3 months. Consequently, the potential long-term impact of wearables on cardiovascular health outcomes which take time to improve remains inadequately explored. Additionally, health outcomes such as number of calories burned or number of steps taken could not be evaluated: none of the studies included the number of calories burned, and only two included the number of steps in both intervention and control groups.

A strength of the studies included is that most studies included multi-component interventions, such as health education, personal fitness programs, supervised exercise, and low-calorie diets, providing a comprehensive approach to health improvement. The review also included studies with variable health technologies, offering insights into different types of devices.

### Conclusion

The use of wearables in LMICs might be effective in improving health outcomes, such as BMI and DBP, especially when used in conjunction with other health interventions. However, to enhance adoption, there is a need for low-cost and customizable wearables that address specific local needs. It is paramount to better understand the long-term impact of wearables on health outcomes, especially in LMICs if one wants to foster their broader adoption in these regions. As a rapidly growing field of research, it is likely that more studies are being conducted in these regions. However, there is a need to harmonize study designs and study endpoints as well as increasing the sample size and the duration of follow-up so that the long-term impact of wearables with or without additional health interventions can be effectively evaluated.
