# Supplementary material for: Are Wearables Effective in LMICs?
Source: Public Health Rev. 2025 May 9;46:1607940. doi: 10.3389/phrs.2025.1607940 (PMC12098041; doi:10.3389/phrs.2025.1607940)

Supplementary material

# Appendix S1: Search Strategy

## Web of Science

**TS= (**"developing countries" OR "africa" OR "asia" OR "caribbean" OR "west indies" OR "south america" OR "latin america" OR "central america" OR "afghanistan" OR “Albania” OR “Algeria” OR "angola" OR “Argentina” OR "armenia" OR "armenian" OR “Azerbaijan” OR "bangladesh" OR “Belarus” OR “Belize” OR "benin" OR "bhutan" OR "bolivia" OR “Bosnia” OR “Botswana” OR “Brazil” OR “Bulgaria” OR "burkina faso" OR "burkina fasso" OR "burundi" OR “Cabo Verde” OR "cambodia" OR “Cameroon” OR "central african republic" OR "chad" OR “China” OR “Colombia” OR "comoros" OR "congo" OR “Costa Rica” OR "cote d ivoire" OR "ivory coast" OR “Cuba” OR "djibouti" OR “Dominica*” OR “Ecuador” OR "egypt" OR "el salvador" OR "eritrea" OR “Eswatini” OR "ethiopia" OR “Fiji” OR “Gabon” OR "gambia" OR "gaza" OR "georgia" OR "ghana" OR “Grenada” OR "guatemala" OR "guinea" OR "guam" OR “Guyana” OR "haiti" OR "honduras" OR "india" OR "indonesia" OR “Iran” OR “Iraq” OR “Jamaica” OR “Jordan” OR “Kazakhstan” OR "kenya" OR "kiribati" OR "korea" OR "kosovo" OR "kyrgyzstan" OR "lao pdr" OR “Lebanon” OR "lesotho" OR "liberia" OR "madagascar" OR "malawi" OR “Malaysia” OR “Maldives” OR "mali" OR "mauritania" OR “Mauritius” OR “Mexico” OR “Micronesia” OR "moldova" OR "mongolia" OR “Montenegro” OR "morocco" OR "mozambique" OR "myanmar" OR "myanma" OR “Namibia” OR "nepal" OR "nicaragua" OR "niger" OR "nigeria" OR “North Macedonia” OR "pakistan" OR “Palau” OR "paraguay" OR “Peru” OR "philippines" OR "philipines" OR "phillipines" OR "phillippines" OR “Russia*” OR "rwanda" OR "ruanda" OR “Samoa” OR "sao tome" OR "senegal" OR “Serbia” OR “Sierra Leone” OR "sri lanka" OR "solomon islands" OR "somalia" OR “South Africa” OR "sudan" OR "swaziland" OR “Suriname” OR “Syria*” OR "tajikistan" OR "tanzania" OR “Thailand” OR "timor-leste" OR "tokelau" OR "togo" OR “Tonga” OR “Tunisia” OR “Turkey” OR “Turkmenistan” OR "tuvalu" OR "uganda" OR "ukraine" OR "uzbekistan" OR "vanuatu" OR "vietnam" OR "viet nam" OR "west bank" OR "yemen" OR "zambia" OR "zimbabwe" OR "middle income" OR "low income" OR "low gdp" OR "low gross domestic" OR "low gross national" OR "low income countr*" OR "middle income countr*" OR "lmic”OR "lmics" OR "third world" OR "lami countr*" OR "transitional countr*" OR "high burden countr*"OR "high burden countr*" OR "countdown countr*")

**AND**

**TS =(**“wearable*” OR "wearable computer*" OR "wearable electronic device*" OR "wearable device*" OR "wearable technolog*" OR "wearable healthcare" OR "Wearable fitness" OR “Wearable sensor*” OR "Electronic Skin" OR "Fitbit*" OR "health wearable*" OR "smartband*" OR "smartwatch*" OR "fitness tracker*" OR "wearable tracker*" OR "wearable activity tracker*" OR "fitness tracker*" OR "activity tracker*" OR "wrist wearable device*" OR "step count*" OR "Apple Watch" OR "Fitbit Charge 2” OR "passive sensing" OR "photoplethysmography" OR “pedometer*” OR “Accelerometer*” OR “Actigraphy” OR "Remote monitoring")

**AND**

**TS = (**"non communicable diseases*" OR "noncommunicable disease*" OR "NCDs" OR "non infectious disease*" OR "noninfectious disease*" OR "cardiovascular disease*" OR "CVD" OR "Heart Failure" OR "Obesity" OR “Metabolic Syndrome” OR "diabet*" OR "hypertension" OR "high blood pressure" OR "heart disease*" OR "chronic disease*" OR "myocardial ischemia" OR "angina" OR "atrial fibrillation" OR "stroke" OR "brain Ischemia" OR "cerebrovascular accident")

**561 results** on the 21.06.23 for studies in English or French published between 2010-2023

## PubMed

(("Wearable Electronic Devices"[Mesh] OR "Fitness Trackers"[Mesh] OR “wearable*”[Title/Abstract] OR "wearable computer*"[Title/Abstract] OR "wearable electronic device*"[Title/Abstract] OR "wearable device*"[Title/Abstract] OR "wearable technolog*"[Title/Abstract] OR "wearable healthcare"[Title/Abstract] OR "Wearable fitness"[Title/Abstract] OR “Wearable sensor*”[Title/Abstract] OR "Electronic Skin"[Title/Abstract] OR "Fitbit*"[Title/Abstract] OR "health wearable*"[Title/Abstract] OR "smartband*"[Title/Abstract] OR "smartwatch*"[Title/Abstract] OR "fitness tracker*"[Title/Abstract] OR "wearable tracker*"[Title/Abstract] OR "wearable activity tracker*"[Title/Abstract] OR "fitness tracker*"[Title/Abstract] OR "activity tracker*"[Title/Abstract] OR "wrist wearable device*"[Title/Abstract] OR "step count*"[Title/Abstract] OR "Apple Watch"[Title/Abstract] OR "Fitbit Charge 2”[Title/Abstract] OR "passive sensing"[Title/Abstract] OR "photoplethysmography"[Title/Abstract] OR “pedometer*”[Title/Abstract] OR “Accelerometer*”[Title/Abstract] OR “Actigraphy”[Title/Abstract] OR "Remote monitoring"[Title/Abstract]) AND ("Noncommunicable Diseases"[MeSH Terms] OR "Heart Failure"[MeSH Terms] OR "Obesity"[MeSH Terms] OR "Diabetes Mellitus"[MeSH Terms] OR "Heart Diseases"[MeSH Terms] OR "Hypertension"[MeSH Terms] OR "Cardiovascular Diseases"[MeSH Terms] OR "non communicable diseases*"[Title/Abstract] OR "noncommunicable disease*"[Title/Abstract] OR "NCDs"[Title/Abstract] OR "non infectious disease*"[Title/Abstract] OR "noninfectious disease*"[Title/Abstract] OR "cardiovascular disease*"[Title/Abstract] OR "CVD"[Title/Abstract] OR "Heart Failure"[Title/Abstract] OR "Obesity"[Title/Abstract] OR “Metabolic Syndrome”[Title/Abstract] OR "diabet*"[Title/Abstract] OR "hypertension"[Title/Abstract] OR "high blood pressure"[Title/Abstract] OR "heart disease*"[Title/Abstract] OR "chronic disease*"[Title/Abstract] OR "myocardial ischemia"[Title/Abstract] OR "angina"[Title/Abstract] OR "atrial fibrillation"[Title/Abstract] OR "stroke"[MeSH Terms] OR "stroke"[Title/Abstract] OR "brain Ischemia"[Title/Abstract] OR "cerebrovascular accident"[Title/Abstract])) AND (("Developing Countries"[Mesh] OR "developing countries"[Title/Abstract] OR "africa"[Title/Abstract] OR "asia"[Title/Abstract] OR "caribbean"[Title/Abstract] OR "west indies"[Title/Abstract] OR "south america"[Title/Abstract] OR "latin america"[Title/Abstract] OR "central america"[Title/Abstract] OR "afghanistan"[Title/Abstract] OR “Albania”[Title/Abstract] OR “Algeria”[Title/Abstract] OR "angola"[Title/Abstract] OR “Argentina”[Title/Abstract] OR "armenia"[Title/Abstract] OR "armenian"[Title/Abstract] OR “Azerbaijan”[Title/Abstract] OR "bangladesh"[Title/Abstract] OR “Belarus”[Title/Abstract] OR “Belize”[Title/Abstract] OR "benin"[Title/Abstract] OR "bhutan"[Title/Abstract] OR "bolivia"[Title/Abstract] OR “Bosnia”[Title/Abstract] OR “Botswana”[Title/Abstract] OR “Brazil”[Title/Abstract] OR “Bulgaria”[Title/Abstract] OR "burkina faso"[Title/Abstract] OR "burkina fasso"[Title/Abstract] OR "burundi"[Title/Abstract] OR “Cabo Verde”[Title/Abstract] OR "cambodia"[Title/Abstract] OR “Cameroon”[Title/Abstract] OR "central african republic"[Title/Abstract] OR "chad"[Title/Abstract] OR “China”[Title/Abstract] OR “Colombia”[Title/Abstract] OR "comoros"[Title/Abstract] OR "congo"[Title/Abstract] OR “Costa Rica”[Title/Abstract] OR "cote d ivoire"[Title/Abstract] OR "ivory coast"[Title/Abstract] OR “Cuba”[Title/Abstract] OR "djibouti"[Title/Abstract] OR “Dominica*”[Title/Abstract] OR “Ecuador”[Title/Abstract] OR "egypt"[Title/Abstract] OR "el salvador"[Title/Abstract] OR "eritrea"[Title/Abstract] OR “Eswatini”[Title/Abstract] OR "ethiopia"[Title/Abstract] OR “Fiji”[Title/Abstract] OR “Gabon” [Title/Abstract] OR "gambia"[Title/Abstract] OR "gaza"[Title/Abstract] OR "georgia"[Title/Abstract] OR "ghana"[Title/Abstract] OR “Grenada”[Title/Abstract] OR "guatemala"[Title/Abstract] OR "guinea"[Title/Abstract] OR "guam"[Title/Abstract] OR “Guyana”[Title/Abstract] OR "haiti"[Title/Abstract] OR "honduras"[Title/Abstract] OR "india"[Title/Abstract] OR "indonesia"[Title/Abstract] OR “Iran”[Title/Abstract] OR “Iraq”[Title/Abstract] OR “Jamaica” [Title/Abstract] OR “Jordan”[Title/Abstract] OR “Kazakhstan”[Title/Abstract] OR "kenya"[Title/Abstract] OR "kiribati"[Title/Abstract] OR "korea"[Title/Abstract] OR "kosovo"[Title/Abstract] OR "kyrgyzstan"[Title/Abstract] OR "lao pdr"[Title/Abstract] OR “Lebanon”[Title/Abstract] OR "lesotho"[Title/Abstract] OR "liberia"[Title/Abstract] OR "madagascar"[Title/Abstract] OR "malawi"[Title/Abstract] OR “Malaysia”[Title/Abstract] OR “Maldives”[Title/Abstract] OR "mali"[Title/Abstract] OR "mauritania"[Title/Abstract] OR “Mauritius”[Title/Abstract] OR “Mexico”[Title/Abstract] OR “Micronesia”[Title/Abstract] OR "moldova"[Title/Abstract] OR "mongolia"[Title/Abstract] OR “Montenegro”[Title/Abstract] OR "morocco"[Title/Abstract] OR "mozambique"[Title/Abstract] OR "myanmar"[Title/Abstract] OR "myanma"[Title/Abstract] OR “Namibia” [Title/Abstract] OR "nepal"[Title/Abstract] OR "nicaragua"[Title/Abstract] OR "niger"[Title/Abstract] OR "nigeria"[Title/Abstract] OR “North Macedonia” [Title/Abstract] OR "pakistan"[Title/Abstract] OR “Palau”[Title/Abstract] OR "paraguay"[Title/Abstract] OR “Peru”[Title/Abstract] OR "philippines"[Title/Abstract] OR "philipines"[Title/Abstract] OR "phillipines"[Title/Abstract] OR "phillippines"[Title/Abstract] OR “Russia*”[Title/Abstract] OR "rwanda"[Title/Abstract] OR "ruanda"[Title/Abstract] OR “Samoa” [Title/Abstract] OR "sao tome"[Title/Abstract] OR "senegal"[Title/Abstract] OR “Serbia”[Title/Abstract] OR “Sierra Leone”[Title/Abstract] OR "sri lanka"[Title/Abstract] OR "solomon islands"[Title/Abstract] OR "somalia"[Title/Abstract] OR “South Africa”[Title/Abstract] OR "sudan"[Title/Abstract] OR "swaziland"[Title/Abstract] OR “Suriname”[Title/Abstract] OR “Syria*”[Title/Abstract] OR "tajikistan"[Title/Abstract] OR "tanzania"[Title/Abstract] OR “Thailand”[Title/Abstract] OR "timor-leste"[Title/Abstract] OR "tokelau"[Title/Abstract] OR "togo"[Title/Abstract] OR “Tonga”[Title/Abstract] OR “Tunisia”[Title/Abstract] OR “Turkey”[Title/Abstract] OR “Turkmenistan”[Title/Abstract] OR "tuvalu"[Title/Abstract] OR "uganda"[Title/Abstract] OR "ukraine"[Title/Abstract] OR "uzbekistan"[Title/Abstract] OR "vanuatu"[Title/Abstract] OR "vietnam"[Title/Abstract] OR "viet nam"[Title/Abstract] OR "west bank"[Title/Abstract] OR "yemen"[Title/Abstract] OR "zambia"[Title/Abstract] OR "zimbabwe"[Title/Abstract] OR (("developing"[Title/Abstract] OR "less developed"[Title/Abstract] OR "under developed"[Title/Abstract] OR "underdeveloped"[Title/Abstract] OR "middle income"[Title/Abstract] OR "low income"[Title/Abstract]) AND ("economy"[Title/Abstract] OR "economies"[Title/Abstract])) OR "low gdp"[Title/Abstract] OR "low gross domestic"[Title/Abstract] OR "low gross national"[Title/Abstract] OR "low income countr*"[Title/Abstract] OR "middle income countr*"[Title/Abstract] OR "lmic"[Title/Abstract] OR "lmics"[Title/Abstract] OR "third world"[Title/Abstract] OR "lami countr*"[Title/Abstract] OR "transitional countr*"[Title/Abstract] OR "high burden countr*"[Title/Abstract] OR "high burden countr*"[Title/Abstract] OR "countdown countr*"[Title/Abstract]))

**Filters: 2010-2023; Full-text and Abstract available; English and French**

**329 results on 21.06.2023**

# Table S2: Results on five different outcomes measures as reported by individual study and calculated as a mean change from baseline

| **Author, Year** | **Reported Results** | | | | | **Calculated Change-from-baseline (Mean ± SD)** | | | |
| --- | --- | --- | --- | --- | --- | --- | --- | --- | --- |
|  | **BMI** | **HbA1c** | **SBP** | **DBP** | **Number of steps/day** | **BMI** | **HbA1c** | **SBP** | **DBP** |
| **Cayir, 2015** | **Baseline: mean** ± **SD**  I: 35.7 ± 2.3  C: 34.5 ± 2.8  **3^rd^ month: mean** ± SD  I: 32.1 ± 2.7  C: 33.7 ± 2.7 |  |  |  | **Baseline: mean** ± **SD**  I: 8817 ± 2725  **3^rd^ month: mean** ± **SD**  I: 9716 ± 2811 | I: -3.6 ± 1.2  C: -0.1 ± 0.7 |  |  |  |
| **Li, 2021** | **Change after 3 months: median (IQR)**  I: –0.60 (–1.07 to 0.16)  C: –0.32 (–0.74 to 0.01) | **Change after 3 months: median (IQR)**  I: –0.55 (–1.53 to –0.07)  C: –0.70 (–1.40 to 0.40) |  |  |  | I: -0.6 ± 0.91  C: -0.32 ± 0.56 | I: -0.55 ± 1.08  C: -0.7 ± 1.33 |  |  |
| **Omar, 2023** | **Baseline: Mean** ± **SD**  I: 26.13 ± 5.99  C: 24.49 ± 4.54  **At 12 weeks: Mean** ± **SD**  I: 25.43 ± 5.27  C: 24.54 ± 4.57 |  | **Baseline: Mean**± **SD**  I: 120.22 ± 8.97  C: 122.12 ± 8.23  **At 12 weeks: Mean**± **SD**  I: 116.3 ± 9.62  C: 118.71 ± 10.63 | **Baseline: Mean**± **SD**  I: 64.70 ± 8.84  C: 67.52 ± 8.31  **At 12 weeks: Mean**± **SD**  I: 63.83 ± 8.73  C: 67.82 ± 6.68 | **Baseline: Mean**± **SD**  I: 4,996 ± 805  C: 4,983 ± 366  **At 12 weeks: Mean**± **SD**  I: 10,128 ± 511  C: 5,697 ± 407 | I: -0.7 ± 1.33  C: 0.05 ± 1.1 |  | I: -3.89 ±2.19  C: -3.41 ± 2.3 | I: -0.87 ± 2.07  C: 0.3 ± 1.83 |
| **Chongthawonsatid, 2017** | **Baseline:**  **mean ± SD**  I: 26.93 ± 0.75  C: 25.86 ± 0.80  **3^rd^ month: Mean** ± **SD**  I: 26.78 ± 0.72  C: 25.62 ± 0.77 |  | **Baseline: Mean**± **SD**  I: 131.40 ± 1.72 C: 135.19 ± 1.84  **3^rd^ month: Mean**± **SD**  I: 125.63 ± 2.02 C: 128.39 ± 2.17 | **Baseline: Mean**± **SD**  I: 82.57 ± 7.91 C:78.77 ± 8.37  **3^rd^ month: Mean**± **SD**  I: 76.40 ± 7.64 C: 74.54 ± 11.26 |  | I: -0.15 ± 0.19  C: -0.24 ± 0.218 |  | I: -5.77 ± 0.48  C: -6.8 ± 0.56 | I: -6.17 ± 2.01  C: -4.23 ± 2.75 |
| **Shenoy, 2010** | **Pre-training: Mean**± **SD**  I: 27.6 ± 2.9 C: 26.3 ± 1.5  **Post-training (8 weeks): Mean** ± **SD**  I: 26.5 ± 2.8 C: 26.9 ± 1.5 | **Pre-training: Mean**± **SD**  I: 7.25 ± 1 C: 7.55 ± 0.91  **Post-training (8-weeks): Mean**± **SD**  I: 6.5 ± 0.87 C: 7.5 ± 0.90 | **Pre-training: Mean**± **SD**  I: 122 ± 13.8 C: 131 ± 12.7 **Post-training (8-weeks): Mean**± **SD**  I: 123.5 ± 5.2 C: 127.7 ± 5.2 | **Pre-training: Mean**± **SD**  I: 85.6 ± 16.1 C: 86.0 ± 7.2 **Post-training (8-weeks): Mean**± **SD**  I: 74.8 ± 4.0 C: 82.3 ± 5.1 | **Pre-training: Mean**± **SD**  I: 3291 ± 231  **Post-training (8-weeks)**  I: 4277 ± 168 | I: -1.1 ± 2.85  C: 0.6 ± 1.5 | I: -0.75 ± 0.94  C: -0.05 ± 0.91 | I: 1.3 ± 7.7  C: -3.3 ± 19.6 | I: -10.8 ± 3.99  C: -3.7 ± 1.37 |
| **Timurtas, 2022** |  | **Change-from baseline after 12-weeks**  I: −0.5 (1.8)  C: −0.9 (0.9) |  |  |  |  | I: -0.5 ± 1.8  C: -0.9 ± 0.9 |  |  |
| **Arovah, 2018** |  | **Baseline: *Mean (95% CI)***  I: 7.85 [6.92, 8.79] C: 7.75 [6.76, 8.73]  **Week 12**  I: 7.43 [6.72, 8.15] C: 7.31 [6.66, 7.95]  **Week 24**  I: 7.24 [6.67, 7.8]  C: 7.22 [6.56, 7.88] |  |  | **Baseline: *Mean (95% CI)***  I: 4876 [3975, 5778] C: 4625 [3776, 5473]  **Week 12**  I: 8096 [6901, 9292] C: 6027 [4835, 7219]  **Week 24**  I: 8214 [6878, 9550]  C: 5898 [4630, 7166] |  | **Week 12**  I: -0.42 ± 1.86  C: -0.44 ± 1.91  **Week 24**  I: -0.61± 1.86  C: 0.53 ± 1.91 |  |  |
| **Gu Y, 2020** |  |  | **Change-from-baseline at 3 months: Mean (95% CI)**  I: −5.4 (−10.5 to 0.3)  C: −1.4 (−6.3 to 3.4)  **Change-from-baseline at 12 months:**  I: −1.8 (−7.1 to 3.5)  C: 0.8 (−4.4 to 6.1) | **Change-from-baseline at 3 months: Mean (95% CI)**  I: −1.8 (−4.7 to 1.0)  C: −3.1 (−6.0 to −0.3)  **Change from baseline at 12 months:**  I: −2.0 (−4.4 to 0.4)  C: 1.8 (−0.6 to 4.2) |  |  |  | I: -5.4 ± 17.98  C: -1.4 ± 16.15  **12 months**  I: -1.8 ± 17.65  C: 0.8 ± 17.48 | I: -1.8 ± 9.5  C: -3.1 ± 9.5  **12 months**  I:  -2.0 ± 7.99  C: 1.8 ± 7.99 |
| **Yuting, 2023** |  |  | **Change-from baseline at 12 weeks: Mean (SD)**  I: -8.52 (19.73)  C: -1.25 (12.47) | **Change-from baseline at 12 weeks: Mean (SD)**  I: -0.42 (10.91)  C: -0.01(7.19) |  |  |  | I: -8.52 ± 19.73  C: -1.25 ±12.47 | I: -0.42 ± 10.91  C: -0.01 ± 7.19 |

# Table S3: Risk-of-bias visualization for the nine RCTs

Risk-of-bias plots created using *robvis* tool created by McGuinness, LA, Higgins, JPT. Risk-of-bias VISualization (robvis): An R package and Shiny web app for visualizing risk-of-bias assessments. Res Syn Meth. 2020; 1-7. Available at: https://doi.org/10.1002/jrsm.1411
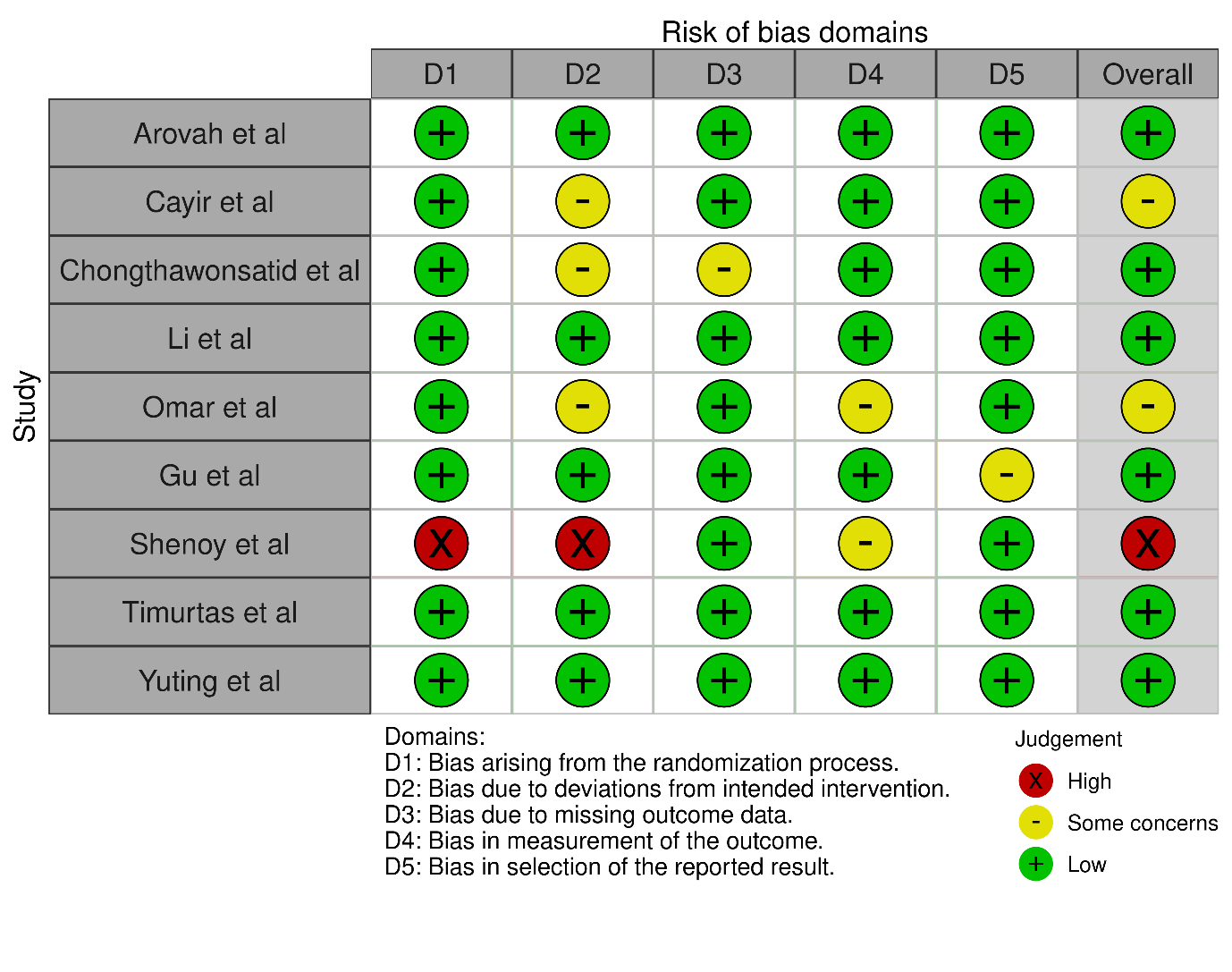

Supplement: Supplementary file 1 [file DataSheet1.docx]
